# Sex differences in Guillain Barré syndrome, chronic inflammatory demyelinating polyradiculoneuropathy and experimental autoimmune neuritis

**DOI:** 10.3389/fimmu.2022.1038411

**Published:** 2022-12-09

**Authors:** Pamela A. McCombe, Todd A. Hardy, Robert J. Nona, Judith M. Greer

**Affiliations:** ^1^ Centre for Clinical Research, The University of Queensland, Brisbane, QLD, Australia; ^2^ Department of Neurology, Concord Hospital, University of Sydney, Sydney, NSW, Australia; ^3^ Brain & Mind Centre, University of Sydney, Sydney, NSW, Australia

**Keywords:** Guillain Barré syndrome, chronic inflammatory demyelinating polyradiculoneuropathy, sex differences, gangliosides, IgG4 antibodies, experimental autoimmune neuritis

## Abstract

Guillain Barré syndrome (GBS) and its variants, and chronic inflammatory demyelinating polyradiculoneuropathy (CIDP and its variants, are regarded as immune mediated neuropathies. Unlike in many autoimmune disorders, GBS and CIDP are more common in males than females. Sex is not a clear predictor of outcome. Experimental autoimmune neuritis (EAN) is an animal model of these diseases, but there are no studies of the effects of sex in EAN. The pathogenesis of GBS and CIDP involves immune response to non-protein antigens, antigen presentation through non-conventional T cells and, in CIDP with nodopathy, IgG4 antibody responses to antigens. There are some reported sex differences in some of these elements of the immune system and we speculate that these sex differences could contribute to the male predominance of these diseases, and suggest that sex differences in peripheral nerves is a topic worthy of further study.

## Introduction

The Guillain Barré syndrome (GBS) and its variants, and chronic inflammatory demyelinating polyradiculoneuropathy (CIDP and its variants, are regarded as immune mediated neuropathies, due to their pathological findings and their response to immune therapy (1–3). Because there is sexual dimorphism of the immune system (4) it could be that the sex of the subject might affect the features of these diseases. This review summarizes knowledge of the effects of sex on GBS and CIDP and discusses possible mechanisms. Firstly, however, we provide a brief overview of GBS and CIDP, and also of their animal model, experimental autoimmune neuritis (EAN).

## Overview of GBS, CIDP and EAN.

### GBS and variants

The term GBS arises from the initial description by Guillain, Barre and Strohl of a syndrome of ascending weakness, associated with loss of deep tendon reflexes, and early recovery, in two soldiers (5). However, the earliest report is likely to be that that of Landry who, in 1859, described a patient who died of respiratory failure after developing ascending weakness (6). The modern use of the term “the Guillain Barre syndrome” is attributed to Haymaker and Kernohan (7). The history of GBS has been written at different times and these reports show evolution of our understanding and conception of the syndrome and its causes (8–10).

Although at one stage GBS was well-known as a demyelinating disease, in modern times it is recognized that there are variants with axonal pathology; it is also known that there are focal variants, such as the Fisher syndrome, first described in 1954 (11). There are clinical and neurophysiological criteria for the diagnosis of GBS (12–14). The “Brighton criteria” (15) are widely accepted for the diagnosis of GBS and Fisher syndrome and are shown in **Table 1**. The prevalence of GBS increases with age up to 70–75 years, then declines (16–18).

**Table 1:** The Brighton Criteria for diagnosis of GBS and Fisher syndrome.

Clinical case definitions: Guillain–Barré syndrome (GBS)
*Level 1 of diagnostic certainty*
Bilateral AND flaccid weakness of the limbs *AND*
Decreased or absent deep tendon reflexes in weak limbs *AND*
Monophasic illness pattern^10^ AND interval between onset and nadir of weakness between 12 h and 28 days AND subsequent clinical plateau *AND*
Electrophysiologic findings consistent with GBS *AND*
Cytoalbuminologic dissociation (i.e., elevation of CSF protein level above laboratory normal value AND CSF total white cell count <50 cells/μl) *AND*
Absence of an identified alternative diagnosis for weakness
*Level 2 of diagnostic certainty*
Bilateral AND flaccid weakness of the limbs *AND*
Decreased or absent deep tendon reflexes in weak limbs *AND*
Monophasic illness pattern AND interval between onset and nadir of weakness between 12 h and 28 days AND subsequent clinical plateau *AND*
CSF total white cell count <50 cells/μl (with or without CSF protein elevation above laboratory normal value) *OR*
IF CSF not collected or results not available, electrophysiologic studies consistent with GBS *AND*
Absence of identified alternative diagnosis for weakness
*Level 3 of diagnostic certainty*
Bilateral AND flaccid weakness of the limbs *AND*
Decreased or absent deep tendon reflexes in weak limbs *AND*
Monophasic illness pattern AND interval between onset and nadir of weakness between 12 h and 28 days AND
subsequent clinical plateau *AND*
Absence of identified alternative diagnosis for weakness
***Clinical case definitions: Fisher syndrome (FS)***
*Level 1 of diagnostic certainty*
Bilateral ophthalmoparesis AND bilateral reduced or absent tendon reflexes, AND ataxia *AND*
Absence of limb weakness *AND*
Monophasic illness pattern AND interval between onset and nadir of weakness between 12 h and 28 days AND subsequent clinical plateau *AND*
Cytoalbuminologic dissociation (i.e., elevation of cerebrospinal protein above the laboratory normal AND total CSF white cell count <50 cells/μl]) *AND*
Nerve conduction studies are normal, OR indicate involvement of sensory nerves only*AND*
*No* alterations in consciousness or corticospinal tract signs *AND*
Absence of identified alternative diagnosis.
*Level 2 of diagnostic certainty*
Bilateral ophthalmoparesis AND bilateral reduced or absent tendon reflexes AND ataxia *AND*
Absence of limb weakness *AND*
Monophasic illness pattern AND interval between onset and nadir of weakness between 12 h and 28 days AND subsequent clinical plateau *AND*
Cerebrospinal fluid (CSF) with a total white cell count <50 cells/μl])(with or without CSF protein elevation above laboratory normal value) *OR*
Nerve conduction studies are normal, OR indicate involvement of sensory nerves only *AND*
*No* alterations in consciousness or corticospinal tract signs *AND*
Absence of identified alternative diagnosis
*Level 3 of diagnostic certainty*
Bilateral ophthalmoparesis AND bilateral reduced or absent tendon reflexes AND ataxia *AND*
Absence of limb weakness *AND*
Monophasic illness pattern AND interval between onset and nadir of weakness between 12 h and 28 days AND subsequent clinical plateau *AND*
*No* alterations in consciousness or corticospinal tract signs *AND*
Absence of identified alternative diagnosis

Based on clinical findings and electrodiagnostic studies, GBS can be divided into subtypes (19). These include acute inflammatory demyelinating polyradiculoneuropathy (AIDP), in which there is inflammation in nerves and demyelination of nerve fibers, acute motor axonal neuropathy (AMAN), and acute motor and sensory neuropathy (AMSAN) which cause generalized weakness with demyelinating or axonal features. The relationship between the demyelinating and axonal disorders is unclear in that there appear to be conditions where there is primary damage to axons, and conditions where severe demyelinating disease leads on to axonal damage (20). There can be difficulty in distinguishing between conduction block and axonal degeneration (21, 22). There are other GBS variants with specific restricted phenotypes; as well as Fisher syndrome (FS), a syndrome of ophthalmoplegia, ataxia and areflexia (23), there is a phenotype of isolated facial diplegia (24) and a pharyngeal-cervical-brachial variant (25).

GBS often follows a preceding illness or event such as an infection. In an analysis of the first 1000 patients enrolled in the International GBS Outcome Study, 72% of patients reported an antecedent infection (26). In that study, the organisms that lead to the preceding infections included *Campylobacter jejuni* (30%), *Mycoplasma pneumoniae* (10%) and cytomegalovirus (4%) (26, 27) and more than one preceding infection was identified in 6% of patients. In our own study, we found that 75% of subjects had a preceding infection (16). This association with a prior infection has led to the view that the immune abnormalities in GBS are triggered in many by exposure to an infectious agent.

However, it must be noted that these infections do not lead to GBS in most people, which suggests that humans vary in their response to infection and in the tendency to develop GBS. It is likely that this is due to genetically mediated variation in the function of the immune system, but it could also be due to “target organ resistance” to immune attack.

There is also increasing recognition that GBS can follow trauma or surgery. The first report of GBS after surgery was in 1968 (28), and since then there have been numbers of case reports. More detailed studies have attempted to quantify the risk. A study from Switzerland found that the relative risk of developing GBS in the 6 weeks after surgery was 13.1 times that of the non-surgically exposed population (29). A French nationwide case-crossover study found that among 8364 cases of GBS, 175 had undergone surgery within the preceding 60 days which increased the risk by greater than 1.5 times compared those who had undergone surgery in the preceding 336-425 days (30). While any recent surgery was a risk, the risk was stronger with bone and digestive organ surgery. Furthermore, a systematic review of 136 cases and 6 cases series of trauma-related GBS found that 89% of patients developed GBS following injury or surgery, with spontaneous intracranial haemorrhage making up nearly 10% of the remaining cases (31). In this study, trauma or surgery to brain and spine were the most likely to pre-date GBS. At present the mechanism for “trauma-related GBS” is speculative and warrants further research.

In AIDP, the inflammatory demyelinating form of GBS, the pathological findings include many macrophages and small numbers of T cells in the nerves (32, 33). Both CD4^+^ and CD8^+^ T cells have been found in nerve biopsies from AIDP patients, as have γδ-T cells and NK T cells (34). Autopsy studies show that pathological changes are prominent in the nerve roots (32). This infiltration is followed by segmental demyelination, meaning demyelination of internodes, which are produced by a single Schwann cell. Demyelination occurs by stripping of myelin by macrophages (35–37). Ultrastructural studies indicate the presence of vesicular degeneration in the outer myelin layer can predate macrophage infiltration (32). Secondary axonal degeneration, thought to occur with severe inflammation, can be seen in spinal roots and in motor and sensory nerves (38). In GBS there are reports of deposition of complement in peripheral nerves (32, 33), including on the outer surface of Schwann cells. The process of recovery from GBS involves remyelination with the production of thinly myelinated fibers with short internodes (37).

In AMAN there is axonal injury but macrophage-activated demyelination and inflammatory infiltrates are not characteristic (39). Activated complement deposition occurs at the nodes of Ranvier with formation of the membrane attack complex and, in addition, macrophages infiltrate along the periaxonal space leading to myelin detachment (39, 40). The resulting injury leads to nodal lengthening and axonal degeneration (39, 41). The pathology of FS, on the other hand, is not well elucidated as it is rarely fatal, and post-mortem studies are rare but the available pathology indicates demyelination of the extraocular nerves (42).

There are many reports of immune abnormalities in GBS and its variants. There have been reports of T cell and antibody reactivity to myelin antigens (eg P0 and P2 proteins) (43), but these were found only in a few patients. These antigens were of interest because immunization of experimental animals with such antigens and adjuvant led to experimental autoimmune neuritis, which shares clinical and histological features with GBS. More recently, immunity to nodal antigens (44) has been demonstrated in GBS.

However, there is now extensive evidence to suggest that glycolipid antigens are the target of a humoral and cell-mediated immune response in some patients with GBS and its variants; this is seen predominantly in AMAN and FS rather than in AIDP and AMSAN (45, 46). The glycolipid targets are mainly gangliosides which are found widely in cell membranes and are important in the biology of the nervous system (47). As well as reactivity to structures found on individual gangliosides there is also reactivity to ganglioside complexes (48). This immune response is thought to be triggered with cross-reactivity between glycolipids on infectious agents and on peripheral nerve. This is known as molecular mimicry (49, 50) and GBS is an excellent example (51). It must be noted that although the gangliosides that are the targets of these antibodies are expressed in other tissues such as the brain and the kidney, the pathological process in GBS is generally confined to the peripheral nerve, suggesting that there is some factor that makes the peripheral nerves vulnerable. However, there are exceptions; GBS can be associated with nephrotic syndrome (52) and with papilloedema (53).

It is notable that the immune response to glycolipid antigens requires antigen presentation by major histocompatibility complex (MHC)-like molecules. These include cluster of differentiation 1 (CD1) and possibly Major Histocompatibility Complex, Class I-Related (MR1) protein (54–57). The T cells that respond to such molecules presented by CD1 or MR1 are sometimes referred to as “unconventional T cells” (58, 59) and include natural killer T (NKT) cells, γδ T cells, and MR1-expressing T cells (MR1T), a subset of which are the mucosal associated invariant T (MAIT) cells (60). Antigen presentation by MAIT cells is potentially relevant to GBS after *C.jejuni* infection since it is a gut infection. Activation of antibody producing cells by NKT cells can occur independently of lymphoid follicles and can lead to a transient activation of plasmablasts (61); this would be consistent with the acute self-limited nature of GBS.

Immune abnormalities are reported in the blood of patients with GBS. These include alterations in levels of T cells The percentage of CD8+ cells in the blood is increased (62) and the proportion of Treg cells is decreased (62, 63). There are increased levels of activated T cells (62, 63).

There are elevated circulating levels of cytokines such as tumor necrosis factor (TNF), interferon (IFN)-γ, interleukin (IL)-1β, IL-6 and IL-17 (64), whereas levels of other circulating factors such as brain derived neurotrophic factor (BDNF) are reduced (65). Stimulation of peripheral blood mononuclear cells (PBMCs) with *C. jejuni* results in a γδT cell response in patients with GBS, and patients with GBS including those with prior *C. jejuni* infection have been found to have increased numbers of circulating γδT cells (66).

In the early stages of GBS infiltrating T cells produce TNFα which can induce demyelination by a direct cytotoxic effect on myelin and by altering myelin protein and glycolipid synthesis (67). Another cytokine of importance is the proinflammatory cytokine IFNα. Th1 cells produce IFNα which activates endothelial cells and enhances expression of major histocompatibility complex (MHC) II leading to an increase in antigen presentation by macrophages. IFNα also effects T cells causing a switch to a Th1 phenotype and stimulating T cell apoptosis. IFNα facilitates TNFα`, IL1β and IL6 production and B cell class switching. Other cytokines implicated in the immunopathogenesis of GBS include IL-17 and IL-23 (68). There is also dysregulation of the IL33/ST2 immune axis (69). A role for Th17 cells is supported by a study showing elevated levels of cytokines of the Th17 pathway (IL17, IL-6 and Il-22) in the cerebrospinal fluid (CSF) of patients with GBS compared to controls (70).

GBS is not thought to be an inherited disease. Some genes have been associated with GBS, but there is no clear HLA association (71). Genetic evidence supports that the killer immunoglobulin receptor (KIR) system is involved in GBS (72); this indicates that the innate immune system is involved in pathogenesis, since KIR molecules are expressed on NK cells that are part of this system (73, 74).

In summary GBS appears to be a monophasic immune mediated disease, associated with antibodies to gangliosides. GBS can be subdivided into groups according to clinical features. GBS patients can also be subdivided according to the serum ganglioside antibody associated with their illness.

### CIDP and variants

CIDP is a term that describes a chronic or relapsing illness, often characterized by demyelination of peripheral nerves and nerve roots. In some patients, CIDP is predominantly a motor neuropathy that causes chronic or recurrent episodes of weakness (75). However, there are variants of CIDP that can show mainly sensory features or focal features (76) and variants that are characterized by axonal degeneration rather than demyelination. These variants include multifocal acquired demyelinating sensory and motor neuropathy (MADSAM), that is characterized by multifocal onset and proximal inflammation of nerve roots and plexuses (77, 78) and distal acquired demyelatinating symmetric (DADS) neuropathy that is characterized by distal but not proximal weakness (77, 79).

Different diagnostic criteria have been developed for the diagnosis of CIDP, with different sensitivity and specificity (76, 80, 81). One challenging issue is the distinction between GBS with fluctuations, and CIDP presenting with an acute deterioration (82, 83). The widely accepted EFNS/PNS criteria (81) provide electrodiagnostic criteria as shown in **Table 2**, and state that:

**Table 2:** EFNS/PNS electrodiagnostic criteria for CIDP.

(1) Definite: at least one of the following
(a) Motor distal latency prolongation >50% above ULN in two nerves (excluding median neuropathy at the wrist from carpal tunnel syndrome), or
(b) Reduction of motor conduction velocity >30% below LLN in two nerves, or
(c) Prolongation of F-wave latency >30% above ULN in two nerves (>50% if amplitude of distal negative peak CMAP <80% LLN values or
(d) Absence of F-waves in two nerves if these nerves have distal negative peak CMAP amplitudes >20% of LLN + >1 other demyelinating parametera in >1 other nerve, or
(e) Partial motor conduction block: >50% amplitude reduction of the proximal negative peak CMAP relative to distal, if distal negative peak CMAP > 20% of LLN, in two nerves, or in one nerve + >1 other demyelinating parametera in >1 other nerve, or
(f) Abnormal temporal dispersion (>30% duration increase between the proximal and distal negative peak CMAP) in >2 nerves, or
(g) Distal CMAP duration (interval between onset of the first negative peak and return to baseline of the last negative peak) increase in >1 nerve (median >6.6 ms, ulnar > 6.7 ms, peroneal > 7.6 ms, tibial > 8.8 ms) + >1 other demyelinating parametera in >1 other nerve
(2) *Probable*
>30% amplitude reduction of the proximal negative peak CMAP relative to distal, excluding the posterior tibial nerve, if distal negative peak CMAP >20% of LLN, in two nerves, or in one nerve + >1 other demyelinating parametera in >1 other nerve
(3) *Possible*
As in (1) but in only one nerve

“*CIDP should be considered in any patient with a progressive symmetrical or asymmetrical polyradiculoneuropathy in whom the clinical course is relapsing and remitting or progresses for more than 2 months, especially if there are positive sensory symptoms, proximal weakness, areflexia without wasting, or preferential loss of vibration or joint position sense.”*

It must be noted that there are other chronic immune-mediated neuropathies, such as multifocal motor neuropathy (84), neuropathy associated with antibodies to myelin-associated glycoprotein (MAG) (85) and Polyneuropathy, Organomegaly, Endocrinopathy, Monoclonal Gammopathy, and Skin Changes Syndrome (POEMS) (86) that are not regarded as CIDP. It is well-known that some neuropathies are associated with circulating paraproteins (87); these include the aforementioned anti-MAG neuropathy and POEMS as well as other neuropathies associated with haematological conditions (88). However, some patients with CIDP also have circulating paraproteins (89). For further information, the reader is referred to more detailed studies of the classification and frequency of CIDP and its variants (90, 91).

In contrast to GBS, there is seldom a history of an infectious illness before the onset of CIDP. A study from Italy found that 15.5% of 435 patients had preceding infections or vaccinations before the onset of disease, and patients with antecedent infections were more likely to have acute onset CIDP with cranial nerve involvement (92). Another large series of 268 patients found preceding infection in 10.4% of subjects (93). Other series reported a frequency of preceding infections ranging from 9.7% of 294 patients (80) to 32% of 92 patients (75).

Findings from sural nerve biopsies indicate that typical CIDP is characterized by paranodal interstitial oedema, and endoneurial cell infiltrates with prominent macrophage invasion causing demyelination (80, 94, 95). Macrophage-mediated damage could facilitate inflammation by exposing new autoantigenic epitopes leading to epitope spreading (96).

In CIDP, in a minority of patients, there have been reports of T cell and antibody reactivity to myelin antigens (43, 97–100). Involvement of cell-mediated immunity in the pathogenesis of CIDP is supported by evidence that activated T cells cross the blood-nerve barrier and that various T cell associated cytokines such as TNFα, IFNα and IL-2 are expressed in abundance in the perineurium, endoneurium and endoneurial blood vessels in patients with CIDP (101). T cells are also found in bopsy samples of nerves from patients with CIDP, and evidence of restricted clonality of CD8+ cells suggests that these cells play a role (96). Both CD4^+^ and CD8^+^ T cells have been found in nerve biopsies from CIDP patients, as have γδ-T cells and NK T cells (34). One study showed that CD8+ cells were more common than CD4+ cells (102). There is also deposition of complement in nerves (103).

In a small minority of patients there are reports of circulating antibodies to periperal nerve, peripheral nerve myelin and peripheral nerve proteins (104). There are rare instances of antibodies to gangliosides GM1 (105) and also LM1, a lacto series ganglioside, in a minority of patients with CIDP (106).

More recently there have been reports of immune reactivity to nodal antigens in a subset of patients with CIDP (44, 107–109). Antibodies targeting these nodal antigens are predominantly of the IgG4 type (107) and the associated syndromes are characterized by poor response to treatment with intravenous immunoglobulin (IVIG). The specific antibody targets in these patients include neurofascin 155, a glial cell paranodal protein, and NF140 and NF186, proteins expressed at nodes and the initial segments of axons (110), and contactin antigens such as contactin-1 (CNTN1) and contactin-associated protein 1 (CASPR1) (111). A comprehensive study of the presence of antibodies in 65 CIDP patients found that 8 had antibodies to nodal/paranodal proteins, 11 had ganglioside antibodies and one had antibody to myelin P2 protein (112). The mechanism of nerve damage from antibodies to nodal/paranodal antigens could involve disruption of axonal-glial junctions (113).

Pathological findings in patients with nodal antibodies are different from those of classical CIDP, without prominent macrophage-mediated demyelination or axonal damage, and this might indicate that nodal/paranodal antibody associated forms of disease have a different pathogenesis to that of classical CIDP. Patients with antibodies to nodal/paranodal antigens are resistant to treatment with IVIG (treatment-resistant CIDP), likely because these antibodies are of the IgG4 type, and are best regarded as having a different disease, called autoimmune nodopathy (AN) (114, 115).

There are some reports of associations of different genes with CIDP, and some reports of human leukocyte antigen (HLA) associations but these have not been replicated in large series (71). There is one report that associations with HLA DR2 are sex dependent, being present in females (116). As with GBS, genetic studies indicated that NK cells of the innate immune system are involved in pathogenesis (117).

In summary CIDP is a chronic or chronic relapsing disease that is immune mediated and associated with a variety of antibodies including gangliosides and nodal and paranodal proteins. Patients with CIDP can be divided into different groups on clinical grounds and also be subdivided according to the antibody they carry.

### Animal models of GBS and CIDP

Animal models of autoimmune disease have been developed since the first description of experimental allergic encephalitis (an animal model of multiple sclerosis) by inoculation with central nervous system tissue and later with the addition of adjuvant, which stimulates the innate immune system (118, 119). The first description of experimental allergic neuritis (EAN), as an animal model of GBS, was provided by Waksman and Adams who set out to produce a disease where inflammation was confined to the peripheral nervous system, and achieved this in rabbits, guinea pigs and mice by inoculation with homogenized nerve (119, 120). EAN was also induced in chickens by inoculation with sciatic nerve (121). EAN can now be induced in many different animal strains with a variety of purified antigens including peripheral myelin proteins, with the use of adjuvants. **Table 3** shows the first descriptions of these models, showing the evolution of types of active EAN and these are discussed below.

**Table 3:** Development of models of actively induced EAN.

Antigen	Animal studied	Reference
**Tissue homogenates**		
Rabbit, bovine, dog, human, guinea pig sciatic nerve, rabbit ganglia	Rabbits	(119, 120)
Guinea pig sciatic nerve	White Leghorn chickens	(121)
Mouse, rabbit, dog, human nerve	White Swiss mice	(119)
Bovine, human, dog nerve	Guinea pigs	(119)
**Peripheral myelin**		
Rabbit peripheral myelin	Rhesus monkeys	(122)
Rat, rabbit, bovine, human, guinea pig peripheral myelin	Lewis rats	(123)
Bovine peripheral myelin	Lewis rats	(124, 125)
Bovine peripheral myelin	Dark Agouti rats	(126)
**Myelin proteins**		
P2 protein	Lewis rats	(127)
P0 protein	Lewis rats	(128)
Peripheral myelin protein 22	Lewis rats	(129)
**Peptides**		
Peptides of P2 protein	Lewis rats	(130, 131)
Peptides of P0 protein	Lewis rats	(132)
Peptides of P0 protein	C57/BL6 mice	(133, 134)
**Protein/lipid complexes**		
P2 protein/phosphatidyl serine complex	Rhesus monkeys	(122)
P2 protein/phosphatidyl serine complex	Lewis rats	(127)
P2 protein/ganglioside complexes	Rabbit	(135)
**Glycolipids**		
Galactocerebroside	Rabbit	(136)
GD1a	Rabbit	(137)
GM1	Rabbit	(138)
GD1b	Rabbit	(136, 139–141)

After induction of EAN by inoculation with peripheral nerve tissue, studies were performed to investigate whether sensitization to myelin could produce disease. EAN was induced with peripheral nerve myelin in rats and monkeys (122, 123, 126) and with bovine peripheral myelin in SJL/J mice (142). These studies were followed by attempts to identify the “ neuritogen” — the component of nerve or myelin that caused the sensitization that led to disease. Myelin P2 protein was the first purified “neuritogen” to be identified (127, 143). Myelin P0 protein can also induce EAN in Lewis rats (128). EAN has also been induced in Lewis rats by inoculation with peripheral myelin protein 22 (PMP22) (129).

Once myelin proteins has been identified as neuritogenic, studies were performed to identify the epitopes of the proteins that caused disease. EAN can be induced by inoculation of P2 peptides in Lewis rats (130, 131). EAN can also be induced in Lewis rats and C57/BL6 mice by inoculation with myelin P0 peptides (132–134). EAN can also be passively transferred by sensitized T cells (144, 145).

These are generally acute forms of EAN. After recovery, animals are resistant to re-induction of disease by inoculation with the same antigen. This acquired tolerance is related to active tolerance by Treg cells. It has been shown that transfer of CD4+CD25+ Treg cells from animals that have recovered from EAN inhibits the induction of EAN (146).

The pathology of EAN is of inflammation and demyelination similar to that of GBS with myelin removal by macrophages (147, 148). There is also deposition of antibody and complement in peripheral nerves (149, 150). Studies of passively transferred EAE suggest a role for T cells in the pathogenesis of disease.

There are few models of CIDP, however immune suppression can convert monophasic EAN induced by peripheral nerve myelin into a chronic form of disease suggesting that in this model, active immune suppression controls the immunity to nerves (151, 152). Chronic EAN can also be induced in Lewis rats by high doses of P0 peptides (132). It can also be induced in Lewis rats by palmitoylated P0 peptides (153).

There is also a model of spontaneous EAN in non-obese diabetic (NOD) mice that are deficient in the co-stimulatory B7-2 molecule (154). In this model there is inflammation and demyelination of peripheral nerves, with a chronic course. However, C57/BL6 mice that overexpress B7-2 also develop spontaneous autoimmune polyneuropathy with a chronic course (155).

Lipids are also major components of myelin. Early studies focused on the possibility that a combination of lipid and protein was required to produce EAN and found that EAN could be induced in rabbits and Lewis rats by inoculation with a complex of lipids and P2 protein (122, 127, 135). Stimulus to finding animal models of disease induced by glycolipids was increased by the finding of antibodies to gangliosides in patients with neuropathy (see above). The first such disease was produced in New Zealand albino rabbits by inoculation with galactocerebroside C (GalC), and is a demyelinating disease (136). Disease induced by GD1a was a flaccid paralysis (137). The disease induced in rabbits by inoculation with GM1 is a severe motor axonal neuropathy (138). The experimental disease produced by GD1b in rabbits is a usually sensory/ataxic neuropathy (136, 139–141) and is associated with apoptosis of cells in the dorsal root ganglion (141).

### Summary of likely pathogenesis of GBS, CIDP and EAN

We now present our views on the pathogenesis of these diseases; we have previously published our views on GBS (156). These diseases are associated with immune changes in peripheral nerve and we take the view that the pathogenesis involves antigen-specific autoimmunity, even though the target antigen is unknown in many cases.

In GBS and CIDP these immune changes can arise after a preceding event, such as an infection, or after a stressful event such as surgery, or can arise without apparent trigger. In the case of the animal models, the immune process is initiated by inoculation. It is likely that host factors play a role in the susceptibility to disease, since not all patients develop GBS after infection, since there are some limited suggestions of genetic associations with GBS and CIDP, and since animal strains vary in susceptibility. It is also notable that there is heterogeneity of GBS and CIDP, both in clinical features and pathology.

It is likely that there is a phase of initiation of disease, when the innate immune system is responsible for activation of the adaptive immune system that leads to an antigen specific process in peripheral nerves and especially the nerve roots. During this phase there is considerable heterogeneity. In GBS after *C. jejuni* infection, the site of initation of innate immunity would be the gut, after respiratory infections antigen would interact with cells in the respiratory mucosa and in EAN, the antigen would be engulfed by dendritic cells in the skin. In EAN, activation of the innate immune system is achieved by the use of adjuvant.

In all cases it is expected that these antigen presenting cells would travel to the regional lymph nodes. In the lymph nodes there would be activation of T cells and B cells. For protein antigens, the interaction between antigen presenting cells and effector cells would require antigen presentation in association with MHC class I and class II molecules (157). For glycolipid antigens, antigen presentation would require CD1 molecules (158). In most cases, activation of T cells and B cells would occur in association with lymph node follicles, but we speculate that in GBS, unconventional extra-follicular activation could occur, since this leads to short term, self-limited immune activation.

The activated cells and antibody then travel though the blood and through the tissues of the body as part of normal immune surveillance and accumulate at the sites where their target antigen is expressed. Antibody also binds to target epitopes and in some cases triggers complement activation.

In the effector phase of disease, demyelination is caused by myelin stripping and other mechanisms other mechanisms. In the axonal disorders, the mechanism of axonal loss includes other mechanisms. There seems to be a role for complement in GBS, CIDP and EAN since complement deposition is seen in biopsies of patients. The role of complement could be to form the membrane attack complex, resulting in damage.

In GBS and acute EAN here is termination of the immune attack- either because the activation was itself limited, such as the transient activation that can occur after infection (159) or because of development of tolerance mechanisms such as Treg cells. In CIDP there is ongoing inflammation/immune processes due to failure of tolerance mechanisms. In the recovery stage of GBS and EAN and during periods of remission of CIDP there is remyelination (seen as thinly myelinated fibers). There is limited capacity for axonal regeneration in GBS and CIDP.

## Sex differences in GBS, CIDP and EAN

### Sex differences in GBS

**Differences in prevalence:** There is evidence that GBS is more common in males than females, and that the incidence increases with age. A systematic review of 63 papers found the incidence of GBS increased with age and was estimated to be between 1.1/100,000 per year to 1.8 per 100,000 per year (17).

A meta-analysis found that the incidence of GBS increases with age and ranges from 0.90 per 100,000 person years in age 20-29 years to 2.66 per 100,000 person years in ages 80-89 years and that at all ages the incidence was greater in males (160); the authors acknowledged that “the reason for the higher risk of GBS in males is unknown”. A large survey of 150,095 GBS patients, part of the global burden of disease (GBD) 2019 project, conducted by the Institute for Health Metrics and Evaluation (IHME), found that the prevalence increased with age until age 75 years and then declined (18) and the prevalence was greater in males than females at all ages up to 75 years.

**Figure 1:**
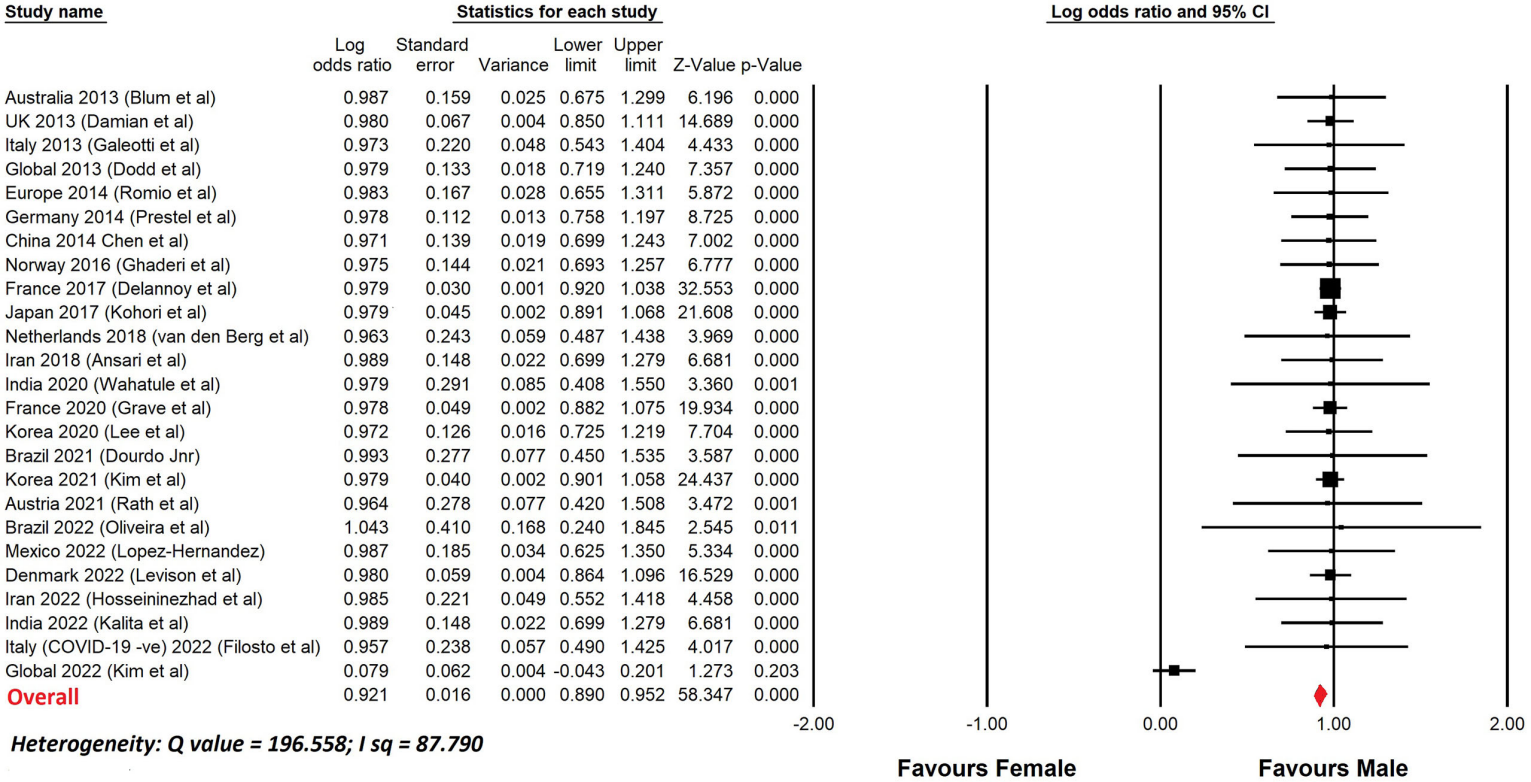
Meta-analysis of sex differences in GBS. This shows a meta-analysis of the data from **Table 4**. Comprehensive Meta-analysis software v 3.31 (Biotech, USA, 2022) was used. Sex differences are displayed using a log odds ratio. The overall odds ratio was 2.7. The overall relative risk was 1.6.

**Table 4** lists a selection of studies of GBS, since 2010, with more than 100 patients, that show the percentage of male patients. In most reports, the number of males was greater than the number of females. **Figure 1** shows a meta-analysis of the data from **Table 4**. The overall odds ratio was 2.7; the overall relative risk was 1.7.

**Table 4:** Percentage of males in GBS series with 100 or more cases since 2010.

Location	year	Number of subjects	% Male	Reference
Australia	2013	335	62%	(16)
UK	2013	1906	56%	(161)
Italy	2013	176	57%	(162)
Global	2013	479	55%	(163)
Europe	2014	303	56%	(164)
Germany	2014	676	55%	(165)
China	2014	441	56%	(166)
Norway	2016	410	55%	(167)
France	2017	9391	58%	(168)
Japan	2017	4132	59%	(169)
Netherlands	2018	144	54%	(170)
Iran	2018	388	62%	(171)
India	2020	100	79%	(172)
France	2020	3523	58.4%	(173)
Korea	2020	533	52%	(174)
Brazil	2021	111	55%	(175)
Korea	2021	5287	58%	(176)
Austria	2021	110	62%	(177)
Brazil	2022	51	65%	(178)
Mexico	2022	248	68%	(179)
Denmark	2022	2414	58%	(180)
Iran	2022	174	60%	(181)
India	2022	388	71%	(182)
Italy (COVID-19 -ve	2022	149	62%	(183)
World	2022	2075	51%	(184)

**Figure 2:**
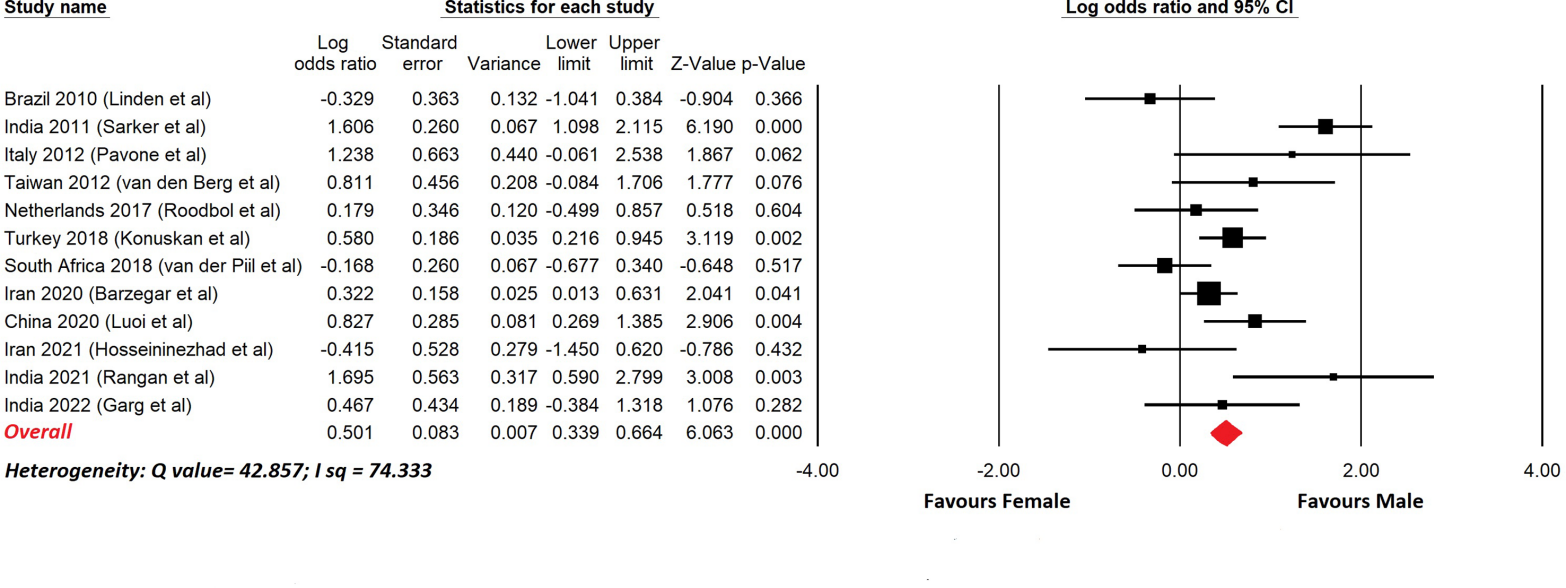
Meta-analysis of sex differences in childhood GBS. This shows a meta-analysis of the data from **Table 5**. Comprehensive Meta-analysis software v 3.31 (Biotech, USA, 2022) was used. Sex differences are displayed using a log odds ratio. The overall odds ratio was 1.6. The overall relative risk was 1.3.

There have been reports of childhood GBS and a selection of these with more than 20 cases are shown in **Table 5**. In most cases the upper age limit was 18 years, so these series included pre- and post-pubertal subjects. This is important because sex differences due to the effects of gonadal hormones become apparent after puberty. **Figure 2** shows a meta-analysis of the data in **Table 5**. The overall odds ratio was 1.7; the overall relative risk was 1.3.

**Table 5:** Percentage of males in childhood GBS series of 20 or more cases since 2010.

Location	Year	Number of subjects	% Male	Reference
Brazil	2010	61	46%	(185)
India	2011	139	69%	(186)
Italy (upper age 14)	2012	20	65%	(187)
Taiwan (up to 18 years)	2012	40	60%	(188)
Netherlands (upper age 16 years)	2017	67	52%	(189)
Turkey (upper age unknown)	2018	236	57%	(190)
South Africa (up to 12 years)	2018	119	48%	(191)
Iran (upper age unknown)	2020	324	54%	(192)
China (upper age 18)	2020	103	60%	(193)
Iran (less than 12 years)	2021	29	45%	(181)
India (upper age 12)	2021	30	70%	(194)
India (upper age 12)	2022	43	56%	(195)

**Sex differences in outcome:** The usual treatment for GBS is intravenous infusion of immunoglobulin (IVIG). IVIG was shown to be at least as effective as (if not superior to) plasma exchange in a randomized controlled trial (196). Plasma exchange also appears to be effective, and may be used as an alternative, but tends to be less well tolerated to completion by patients, and is less straightforward to administer (197). Corticosteroids are no of clear benefit and may even be harmful in GBS (198). In GBS, criteria for admission to the intensive care unit include rapidly progressive respiratory muscle weakness, respiratory distress, severe dysautonomia or dysphagia, or a score >4 on the Erasmus GBS respiratory insufficiency score (EGRIS) (199). EGRIS is a composite score that incorporates time from symptom onset to hospitalization, facial and bulbar weakness, and the severity of muscle weakness at hospital admission (200).

A retrospective study of 121 patients from Austria extending back 20 years calculated that sex was not a predictor of prognosis (201). In addition, there was no difference in the odds of receiving more treatment for GBS according to sex. In a large Japanese study of 4132 patients, multivariate regression indicated that males with GBS were less likely than females to require mechanical ventilation (OR 0.76) (169). Once intubated, male patients with GBS tend to have a poorer outcome than females due to ICU-related complications including pneumonia (202).

### Sex differences in CIDP

**Difference in Prevalence:** An early study found the prevalence of CIDP to be 1/100,000 in South East England (203). In Australia, the prevalence was 1.9 per 100,000 (204)[80]. A higher prevalence of 7.7 per 100,000 was found in Norway (205)[81]. Later studies found prevalence of 4.77 per 100,000 using EFNS/PNS criteria and 1.97 per 100,000 using AAN criteria (206).

As in GBS, the incidence and prevalence of CIDP appears to increase with older age. In a Japanese study, the crude incidence rate of CIDP was 0.06 per 100 000 person years in the age group 0-15 years; 0.40 per 100 000 person years in the age group 15-55 years and 0.73 40 per 100 000 person years among those older than 55 years (207). In a Dutch study, incidence was 17 times as high in those with ages greater than 50 years compared to less than 50 years (208). A systematic review and meta-analysis showed that the age specific prevalence increased steadily from childhood through to old age across 5 different studies (209).

**Figure 3:**
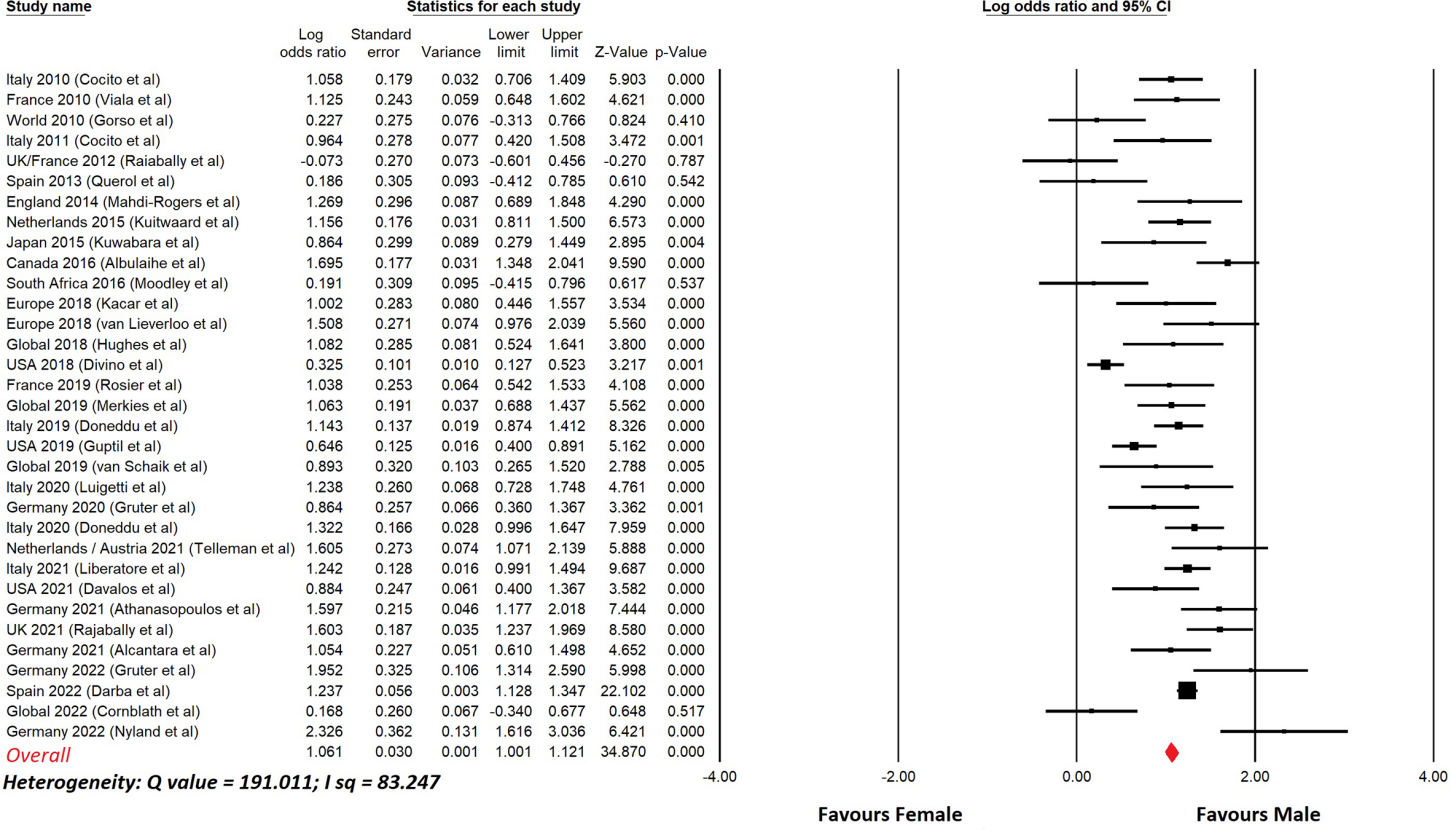
Meta-analysis of sex differences in CIDP.This shows a meta-analysis of the data from **Table 6**. Comprehensive Meta-analysis software v 3.31 (Biotech, USA, 2022) was used. Sex differences are displayed using a log odds ratio. The overall odds ratio was 2.9. The overall relative risk was 1.7.

**Table 6** lists studies of CIDP that show the prevalence in males and females, with nearly all studies showing a male predominance. **Figure 3** shows a meta-analysis of the data in **Table 6**. The overall odds ratio was 2.9; the overall relative risk was 1.7.

**Table 6:** Percentage of males in a selection of studies of CIDP of 80 or more cases since 2010.

Location	Year	Number of subjects	Percent male	Reference
Italy	2010	267	63%	(210)
France	2010	146	64%	(76)
World	2010	106	53%	(211)
Italy	2011	110	62%	(212)
UK/France	2012	110	49%	(213)
Spain	2013	86	52%	(214)
England	2014	101	65%	(215)
Netherlands	2015	281	64%	(216)
Japan	2015	94	61%	(217)
Canada	2016	305	70%	(218)
South Africa	2016	84	52%	(219)
Europe	2018	106	62%	(220)
Europe	2018	125	68%	(221)
World	2018	106	63%	(222)
USA	2018	790	54%	(223)
France	2019	134	63%	(224)
World	2019	235	63%	(225)
Italy	2019	460	64%	(90)
USA	2019	525	58%	(226)
World	2019	82	61%	(227)
Italy	2020	130	65%	(228)
Germany	2020	127	61%	(229)
Italy	2020	323	66%	(92)
Netherlands/Austria	2021	126	69%	(230)
Italy	2021	535	65%	(231)
USA	2021	138	61%	(232)
Germany	2021	203	69%	(233)
UK	2021	268	69%	(93)
Germany	2021	167	63%	(234)
Germany	2022	95	73%	(235)
Spain	2022	2805	65%	(236)
World	2022	119	52%	(237)
Germany	2022	84	76%	(238)

In childhood CIDP, a review of a series of patients combined with a review of previous series found no sex differences (239).

**Sex difference in outcome:** Patients with CIDP can be treated with oral corticosteroids, IVIG or plasma exchange (240). Chronic immune suppressing therapies, such as azathioprine, cyclophosphamide and rituximab are also widely used as part of a combination immune intervention strategy, but quality evidence for efficacy is lacking (241). In the randomized, controlled FORCIDP trial, fingolimod was not of benefit in CIDP (222).

Currently there are trials underway investigating agents that block the neonatal Fc receptor (FcRn) and therefore lead to a decrease in circulating levels of immunoglobulin (Ig) and Ig immune complexes. The results of the first of these trials, which was a phase 2 placebo-controlled trial of rozanoliximab, a monoclonal antibody that antagonizes the FcRn, were disappointing, being negative for an improvement in a patient reported scale of activity and social participation (242).

Sex was not found to be a predictor of severe disability in a multivariate analysis in 165 patients with CIDP (80). A Japanese study found that factors which predict poor responsiveness to IVIG in CIDP patients include male sex, longer disease duration, and slower progression (243).

### Sex differences in animal models of GBS and CIDP

The role of sex differences has not been a focus of studies of EAN. In early studies there was little recognition of the possible importance of sex differences, and the sex of the animal was not reported, only one sex was used or there was no study of sex differences. **Table 3** shows the sex of the animals used in the initial studies of various forms of EAN.

In the original paper, female rabbits were inoculated with CNS tissue and adjuvants (120). In an early paper about EAN in Lewis rats, both sexes were used for inoculation with myelin from guinea pig, frog, rat and rabbit and there was no reported difference between the sexes (123). In another early paper that reported passive transfer of EAN to rabbits, the sex of the donors and the recipients was not stated (244). EAN could be induced in SJL mice by inoculation with myelin but the sex of the mice was not stated (142). In a study of EAN induced in chickens with guinea pig sciatic nerve, both sexes were studied (121). In studies of inoculation of Lewis rats with bovine myelin, male (124) and female Lewis (125) rats have been used.

In the early studies of disease induced in Lewis rats with P2 protein, males were used (127). EAN can be induced in male (130) and female (130, 131) Lewis rats by peptides of myelin P2 protein. Recently there are numbers of studies of EAE induced in female Lewis rats (245–250) by P2 peptides, and occasional studies of males (251) but no studies comparing the sexes.

EAN was induced by myelin P0 protein in male Lewis rats (128). EAN was induced by peptides of P0 protein in male Lewis rats (132). With this model, males have been used in recent studies (252). EAN was induced also with peptides of P0 and in male (133) and female C57/BL6 mice (134), and recent studies have used male Lewis rats (253). PMP22 EAN has been induced in male Lewis rats (129) but there are no recent studies.

Similarly, there is little information about the diseases induced by glycolipids. EAN after GalC sensitization was produced in male New Zealand rabbits (136). GM1 disease and GD1a disease was first induced in male Japanese white rabbits (138, 254, 255). The sex of the rabbits used for induction of GD1 disease was not specified (140).

It must be noted that in experimental autoimmune encephalomyelitis, which has been more thoroughly studied than EAN, and where sex differences have been explored, the effects of sex are complex and vary with animal species/strain and with the inoculating antigen (256).

## Discussion

There are sex differences (sexual dimorphism) in many aspects of physiology and pathophysiology. These have developed since the evolution of the sex chromosomes and sexual reproduction (257–259). Sex differences can be attributed to the effects of the sex chromosomes and to the effects of gonadal hormones. There are widespread examples of sexual dimorphism in morphology, physiology and biochemistry (260). There is marked sexual dimorphism of the immune system (261, 262). Across the animal kingdom, females have a more active immune system (sometimes referred to as having greater immunocompetence) than males (263, 264). **Tables 7**, **8** show some sex differences in the immune system.

**Table 7:** Sexual dimorphism in immune processes.

Immune functions	Difference between males and females
Immunosenescence	Greater in human males (265)
IgM levels	Greater in human females (266, 267)
Allograft rejection	Greater in females (268)
*In vitro* response to mitogens	Greater in females (269, 270)
Resistance to the induction of immune tolerance	Greater in females (271)
Ability to combat infection	Greater in females (272, 273)
Response to vaccination	Greater in human females (274)
Th1 responses	Greater in females (275)
Antigen presentation of peptides	More efficient in female mice (270)
Phagocytosis by neutrophils and macrophages	Greater in female rats (276)

**Table 8:** Sex differences in immune cell numbers and functions.

Immune cell	Difference between males and females
Percentage of lymphocytes in total leukocyte population	Higher in females (277)
CD4+:CD8+ ratio	Greater in females (278, 279)
Percentage of CD4+ T cells	Greater in females (280)
Percentage of CD8+ cells	Greater in males (280)
NK cell numbers	Higher in males (281)
Neutrophils	More activated in females (282)
Macrophage polarization	More M2 polarization in females (283)
Monocyte	Females show greater transcription of inflammatory genes (284)
Treg cell numbers	Increased in males (285)

In this review we have studied sex differences in GBS and CIDP. GBS and CIDP are consistently more common in males than females. The sex association was less obvious in childhood GBS, where some of the subjects would have been pre-pubertal. Sex differences that appear after puberty can be due to the effects of gonadal hormones. It can be noted that in multiple sclerosis, where there is a strong influence of sex on the prevalence of disease in adults, there is no sex difference in childhood before puberty (286). There is little evidence of an effect of sex on the outcome of disease. It must be noted that the majority of studies were cohort studies rather than population based studies, which is a limitation.

The male predominance would be unusual if GBS and CIDP were like other autoimmune diseases, which are usually more common in females (260, 287). For these other autoimmune diseases, the female predominance is often attributed to the stronger immune system of females, **Tables 7**, **8** provide lists of the effects of sex on different immune cells and functions, and shows that, for many functions, the female immune system has stronger responses. Females have greater resistance to infection, greater resistance to the induction of immune tolerance, greater *in vitro* response to mitogens, greater response to vaccination, greater IgM levels and greater Th1 responses (262). The stronger immune responses are thought to have arisen through evolutionary mechanisms allowing females to live longer to protect their offspring (264).

We have considered what is known about the pathogenesis of GBS and CIDP and now speculate on some possible reasons for the sex differences. The pathogenesis involves macrophages, monocytes, CD4+ T cells, B cells, Th17 cells, antibody, complement and cytokines. As outlined in **Tables 7**, **8**, for most of these elements of the immune system, females are thought to have stonger responses. Exceptions are CD8+ cells and NK cells which have increased levels in males and have a possible role in GBS and CIDP (72, 117, 288). Therefore, we have looked for other explanations for the sex differences in GBS and CIDP.

We consider that GBS and CIDP are autoimmune diseases, likely to be due to an antigen-specific immune response, athough for many patients the antigen remains unknown. However, GBS and CIDP have some features that are unusual for autoimmune diseases. Criteria for accepting an autoimmune aetiology for disease were first put forward in 1957 (289) and later updated in 1993 (290). These criteria include direct proof (transmission to another human or experimental animal), indirect proof (induction of disease by autoantigen, pathological features) and circumstantial evidence (MHC association). GBS and CIDP have features that differ from these criteria. There is no clear target antigen – although it could be argued that each of the syndromes associated with the known targets of autoantibodies could be regarded as separate diseases. For some of the known antigen, namely the glycolipid antigens, disease transfer has been more difficult than with more classical autoimmune diseases. Finally, there is no clear HLA association (71); this could be related primarily to the fact that, in GBS, and some CIDP patients, as opposed to the case in well-recognized autoimmune diseases, the target antigens are not proteins but glycolipids, which are not presented by HLA molecules. There is more limited polymorphism in the CD1 and MR1 molecules than for HLA molecules, but nothing is currently known about whether there are any sex-related effects in their expression.

GBS differs from autoimmune diseases in being self-limited. This suggests a transient activation of the immune system. This can occur through activation of plasmablasts by pathways that do not include the lymphoid follicle or through non-canonical pathways of activation (291). There are some suggestions that sex influences the activation of plasmablasts and plasma cells (292). Further exploration of the role of plasmablast activation could be useful in understanding GBS and exploring sex differences.

Genome wide gene expression analysis of peripheral leukocytes has indicated differences between male and female patients with GBS. In one study, male GBS patients were enriched for twenty genes involved in a range of immunological processes, including macrophage and leukocyte migration, and female GBS patients were enriched for 62 genes including those for viral infection and defense (293). Genes involved in the production of matrix metaloproteinase-9 (MMP9), which has previously been shown to be associated with disease severity in GBS, were highly expressed in males implicating MMP9 as being potentially relevant to the higher prevalence of GBS in males.

In most cases of GBS, and some patients with CIDP, gangliosides are the target antigen. Gangliosides are small glycolipid molecules that react with unconventional T cells including γδ T cells, NKT cells and MR1T/MAIT cells (58–60) after antigen presentation in association with CD1 (158). Little is known about sex differences in these pathways.

However, one study measured the numbers of unconventional T cells and showed females had more iNKT cells, fewer γδ T cells and the same number of MAIT cells as males (294). Another study showed that there are sex differences in the function of NKT cells, with male cells having a Th1 bias (295). However, a third study found that women have greater numbers of NKT cells than men, and that stimulation with alpha-galactosylceramide leads to higher production of IFNγ, IL-4, IL-17 and TNF by CD4+ and double negative NKT cells (296). Further studies of the mechanisms of development of reactivity to gangliosides could also be helpful in the understanding of GBS and CIDP and possible sex differences.

In CIDP, studies suggest a role for CD8 cells, that interact with MHC class I. A recent study found that HLA-associated shaping of T cell receptor B variable (TCRBV) usage in CD8^+^ T cells differed between the sexes, with male cells showing greater expansion of TCRBV usage than females (297); this is evidence that there could be sex differences in the capacity of these cells to interact with self antigens. It remains to be determined if this occurs in CIDP.

Patients with “treatment resistant CIDP” can be placed in a separate group of autoimmune nodopathies (AN), which are emediated by IgG4 antibodies. There are other autoimmune diseases associated with IgG4 antibodies, and some of these are neurological, including anti-muscle specific tyrosine kinease (MUSK) myasthenia gravis, leucine-rich glioma-inactivated (LGI)-1 and CASPR2 autoimmune syndromes, and anti-immunoglobulin LSAMP, OBCAM, Neurotrimin 5 (IgLON5) disorder disorder (298). It must be noted, that in contrast to the findings in CIDP, MUSK MG is reported to be more common in females. A switch to IgG4 antibody production is triggered by chronic antigen exposure, and an environment enriched by T cell-produced cytokines, in particular IL-4, IL-10, IL-12, IL-13 and IL-21 (299).There are many sex differences in T cells, for example the ratio of Th1 to Th2 cells is greater for females than males (300)and there are sex differences in the regulation of TfH cells, which are needed for antibody production (301). Interestingly, a group of systemic immune-mediated disorders that are all characterized by infiltration of IgG4-expressing plasma cells into involved organs have now been consolidated into a grouping known as IgG4-related disease, and one of the predominant clinical features is the male predominance of these disorders (302).

In CIDP, another distinguishing feature that could suggest unusual immunological features, is the lack of response to therapies that were expected to be of benefit. This could be due to the acknowledged challenges of trials in CIDP, for example the difficulty in identifying patients with active disease who deteriorate after withdrawal of IVIG. However, some of the pathological features of CIDP are similar to those of multiple sclerosis (MS), but fingolimod, which is of benefit in MS, was not helpful in CIDP. In antibody mediated diseases, such as myasthenia gravis (MG), blockade of the neonatal Fc receptor is of benefit. However, the results of the first trial in CIDP showed no benefit (242). There is no information about what these failures reveal about the underlying pathogenesis of CIDP. However, this could be a further indicator that CIDP differs from other immune and inflammatory diseases.

The sex differences in GBS and CIDP could relate to sex differences in the underlying immunopathogenetic mechanisms. However, our understandng of the pathogenesis of GBS and CIDP is incomplete. Going forward, it will be beneficial to perform further studies of the immunological basis of GBS and CIDP, with attention to the heterogeneity of these diseases. There is marked clinical heterogeneity and also heterogeneity in the immunological targets that have been identified. Once the immunological basis of the subtypes of disease is clear, then further attention can be given to the sex differences in these mechanisms.

It must be noted that the prevalence of GBS and CIDP increases with age. In myasthenia gravis, which shows a late peak of disease (late-onset MG), studies, including our own, have also shown that there is an increased proportion of males in the late onset disease (303, 304). This could suggest that older males are more susceptible to immune disease and this could contribute to the male predominance. It is known that there are changes in the immune system with aging (immunosenescence) (305). We have shown sex differences in immunosenescence, with males showing greater decline (265). Above we have speculated on a role for unconventional T cells; there is no information about the effect of ageing on these cells, or any sex differences in these cells with aging. It is possible that the sex differences in immunosenescence could make males more susceptible- possibly by waning of immunoregulation.

Another possible issue is sex differences in the susceptibility of nerves to immunological attack. In autoimmunity, this can be referred to as “target organ resistance”. Little has been written about this, but some of the earliest studies come from the model of autoimmune thyroiditis that arises in obese strain chickens, and is a model of human Hashimoto’s thyroiditis (306). In this disorder, **i**nherited alterations of thyroid function predispose to spontaneous autoimmune thyroiditis (307, 308). This has been related to viral infection and aberrant expression of MHC class II molecules (309). In experimental autoimmune oophoritis, a model of autoimmune ovarian failure (310), it has been shown that after recovery from oophoritis, animals are resistant to further episodes of disease and that this resistance is a property of the recovered ovaries. When normal ovarian tissue was transplanted under the capsule of recovered ovaries, disease developed only in the transplanted tissue. In experimental autoimmune encephalomyelitis, lack of apoptosis in target tissue leads to increased EAE (311). We have previously suggested that sex differences in target organ resistance could contribute to sex differences in autoimmunity (260).

There is some evidence that alterations in peripheral nerve myelin can lead to inflammation of nerves. This is seen in the reports of inflammation typical of CIDP, or the onset of clinical features of CIDP in people with various types of Charcot Marie Tooth (CMT) disease (312–314). This is highly suggestive that variation in the structure of nerves could possibly lead to vulnerability to inflammation.

The underlying mechanism for putative sex differences in target organ resistance of peripheral nerves would be expected to be due to the biological properties of nerves. There are some reports of sex differences in the peripheral nerves and in the response of peripheral nerve to injury. Gene expression profiles from the dorsal root ganglia (DRG) of rats show that 6% of expressed genes differ according to sex with more genes involved in immunological mechanisms expressed in females than males, and that after injury to the DRG, neural pathways linked to pain also show considerable differences between males and females (315). Furthermore, following sciatic nerve axotomy in mice, males exhibited greater expression of structural cytoskeletal proteins in the regenerating nerve than females. In addition there were sex differences in coding and non-coding mRNAs responsible for other neurotrophic, metabolic and sex chromosome-linked molecular programs (316). It is possible that these sex differences in the biology of peripheral nerves could also contribute to the differences in susceptibility to GBS and CIDP. However, there is still much that needs to be determined.

Our conclusion is that there is evidence that unlike other autoimmune diseases, GBS and CIDP are more common in males. This difference is not likely to have any single cause, and could relate to differnces in the immune system or in the target organ. There are no studies of sex differences in EAN and in future it would be very helpful if such studies were to be performed, although we note that in experimental autoimmune encephalomyelitis (EAE), there are many sex differences that are specific for the strain of animal and the type of disease that is studied (256). Therefore it will be necessary to report all these details. It will also be necessary to report the age of the animal, given that sex differences change over time- becoming more apparent with puberty and showing differences in older age. It has also been found that, in some experiments, the sex of the researcher can influence the results of the study (317, 318), so this should also be recorded.

Going forward we suggest that there is a need to investigate sex differences in GBS and CIDP. For males compared to females the odds ratio for developing GBS and CIDP are 2.7 and 2. 9, respectively. This is a substantial increase in risk, and understanding the mechanisms for this could help understand what is important in pathogenesis. We recommend that all epidemiological studies stratify for age and sex, and that all investigations into the biology of GBS and CIDP also take account of the sex of the subjects. We also recommened that all clinical trials should be stratified according to sex, in case there are sex differences in the response to therapy.

There are some challenges. Studies will need to be increased in size, to provide sufficient power to detect sex differences, and investigators need to consider the effects of sex at different stages of investigation such as recruitment, randomization and analysis (319). However, despite the challenges, research into sex differences has the potential to be rewarding. Sex differences are pervasive in all aspects of biology, and the finding that GBS and CIDP are more common in males deserves further enquiry.

## Author contributions

All authors listed have made a substantial, direct, and intellectual contribution to the work, and approved it for publication.
